# Liver uptake on bone scan for prostate cancer metastatic workup

**DOI:** 10.1002/ccr3.2351

**Published:** 2019-08-04

**Authors:** Alain Mwamba Mukendi

**Affiliations:** ^1^ Urology Department Chris Hani Baragwanath Academic Hospital, University of the Witwatersrand Soweto South Africa

**Keywords:** bone scan, liver uptake, metastatic workup, prostate cancer

## Abstract

This clinical image presents an unusual association of diffuse liver uptake on bone scan and prostate adenocarcinoma. Such association has never been described in the literature. The cause of the hepatic uptake should be identified from thorough history taking, technical factors to anatomical diagnostic workup.

A 53‐year‐old male known hypertensive presented with lower urinary tract symptoms and a prostate‐specific antigen of 13.6 µg/L. There was no history of recent radiopharmaceutical administration. Abdominal examination was unremarkable, and digital rectal examination revealed an enlarged smooth firm prostate. Systematic transrectal ultrasound‐guided biopsy of the prostate was done, and a diagnosis of prostate adenocarcinoma Gleason score of 3 + 3 = 6 (International Society of Urological Pathology [ISUP] 1) was made. Bone scan done as part of metastatic workup showed diffuse liver uptake and found no metastatic bone disease Figure 1. Liver function test, hepatitis testing, and liver ultrasound were all normal.

**Figure 1:**
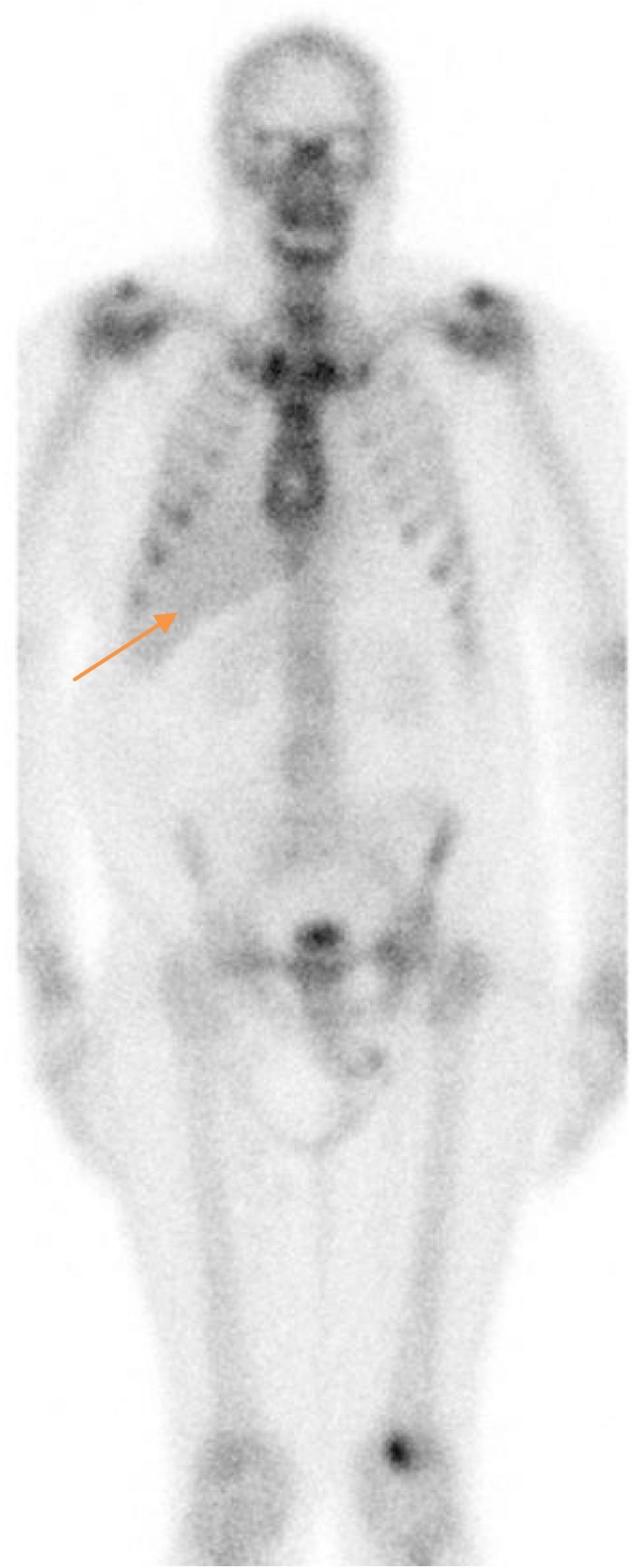
Bone scan image demonstrating no bone metastasis but diffuse liver uptake (orange arrow)

There has never been a description of liver uptake on bone scan associated with prostate adenocarcinoma in the literature.

Hepatic uptake on a bone scan can reflect recent administration of radiopharmaceuticals or liver disorders such as primary or metastatic disease.^1^ Metastatic disease causes focal faint uptake. Diffuse uptake like in this case is unusual, but can be seen with hepatitis, acute hepatic failure, amyloidosis, intravenous contrast medium administration, etc^2^

This is the first reported association of prostate adenocarcinoma and diffuse liver uptake on bone scan. This uptake was nonspecific as no liver pathology was detected on further investigations.

## CONFLICT OF INTEREST

None declared.

## AUTHORS' CONTRIBUTION

AMM: conceived and designed the study, acquired the data, analyzed and interpreted the data, wrote the manuscript, and approved the final manuscript with critical content.

## References

[1] Al‐katib S , Al‐faham Z , Balon H . Liver uptake on bone scanning: a diagnostic algorithm. J Nucl Med Technol. 2015;43:135‐136. 25537759 10.2967/jnmt.114.146449

[2] Verma S , Kumar N , Kheruka SC , Gambhir S . Extraosseous 99mTc‐methylene diphosphonate uptake on bone scan: Unusual scenario. Indian J Nucl Med. 2016;31(4):280‐282. 27833313 10.4103/0972-3919.190799 PMC5041416

